# Insulator-Defect Detection Algorithm Based on Improved YOLOv7

**DOI:** 10.3390/s22228801

**Published:** 2022-11-14

**Authors:** Jianfeng Zheng, Hang Wu, Han Zhang, Zhaoqi Wang, Weiyue Xu

**Affiliations:** 1 School of Mechanical Engineering and Rail Transit, Changzhou University, Changzhou 213164, China; 2 Jiangsu Province Engineering Research Center of High-Level Energy and Power Equipment, Changzhou University, Changzhou 213164, China; 3 Key Laboratory of Noise and Vibration, Institute of Acoustics, Chinese Academy of Sciences, Beijing 100190, China

**Keywords:** YOLOv7, insulator-defect detection, attention mechanism, HorBlock, SIoU

## Abstract

Existing detection methods face a huge challenge in identifying insulators with minor defects when targeting transmission line images with complex backgrounds. To ensure the safe operation of transmission lines, an improved YOLOv7 model is proposed to improve detection results. Firstly, the target boxes of the insulator dataset are clustered based on K-means++ to generate more suitable anchor boxes for detecting insulator-defect targets. Secondly, the Coordinate Attention (CoordAtt) module and HorBlock module are added to the network. Then, in the channel and spatial domains, the network can enhance the effective features of the feature-extraction process and weaken the ineffective features. Finally, the SCYLLA-IoU (SIoU) and focal loss functions are used to accelerate the convergence of the model and solve the imbalance of positive and negative samples. Furthermore, to optimize the overall performance of the model, the method of non-maximum suppression (NMS) is improved to reduce accidental deletion and false detection of defect targets. The experimental results show that the mean average precision of our model is 93.8%, higher than the Faster R-CNN model, the YOLOv7 model, and YOLOv5s model by 7.6%, 3.7%, and 4%, respectively. The proposed YOLOv7 model can effectively realize the accurate detection of small objects in complex backgrounds.

## 1. Introduction

An insulator is a special kind of insulation control and is essential electrical equipment in overhead transmission lines. In early years, insulators were mainly used for power poles and gradually developed into disc insulators, usually made of glass or ceramics, hung on one end of the connection tower of high-voltage power lines. They fill the role of electrical insulation and mechanical fixation in transmission lines [1]. In addition, by hanging insulators on transmission lines, the transmission distance can be increased and capacitive reactance between transmission lines can be reduced. However, due to the long-term influence of factors such as strong electric fields and harsh environments, insulators have many defects, such as self-explosion, damage, pollution flashover, and current leakage [2,3].

Among these defects, the most common faults are insulator damage and surface defects caused by pollution flashovers. The damage is mainly caused by insulator manufacturing defects, the combined action of various stresses, and other reasons. Surface defects are mainly caused by partial discharge in pollution flashovers. Pollution flashover means that pollutants are attached to the insulation surface, and soluble substances gradually dissolve in water under wet conditions, forming a conductive film on the insulation surface that reduces the insulation strength and increases the leakage current, resulting in partial discharge [4]. According to the research, power system paralysis caused by insulator defects accounts for more than half of power grid system failures. Therefore, research on the rapid detection and identification of insulators and their defects has excellent application value for maintenance and repair personnel [5].

The traditional insulator-defect detection method mainly uses helicopter-manned inspection, field inspection, or traditional detection algorithms to analyze pictures taken by robots or drones [6]. However, the structure of China’s transmission lines is complex, and the use of traditional detection methods will lead to a huge waste of financial and material resources, as its efficiency is not high. At the same time, the images taken by uncrewed aerial vehicles have the property of a large field of view. In this scenario, there are some problems with targeting insulator defects, such as a complex background environment and a small defect target, which interfere with defect detection [7]. Therefore, it is urgent to propose a small-target fault detection method for transmission lines with complex backgrounds to improve detection efficiency.

In recent years, with the development of deep-learning theory and the improvement of computer performance, target detection algorithms based on deep learning have been widely studied because of their good generalization ability and cross-scene ability [8]. Zheng et al. [9] proposed a two-stage training method to regularize a depth CNN from the perspective of data attenuation. The experimental results show that the algorithm improves the generalization ability of depth CNNs by optimizing the feature boundary and is robust to the selection of super parameters. Zhao et al. [10] proposed a meteorological photo-classification system based on a multi-channel convolutional neural network (CNN) and improved frame difference method (FDM). The system can work in an embedded system with limited computing resources and can accurately classify cloud observation photos taken by ground cameras. Jin et al. [11] realized deep transfer learning from face recognition to face diagnosis. Successful application of deep transfer learning in face diagnosis with small datasets can provide a low-cost and non-invasive method for disease screening and detection. To replace the depth sensors by generated pseudo-depth maps, Jin et al. [12] also proposed a pseudo-RGB-D face-recognition framework and provided data-driven ways to generate depth maps from 2D face images. Zhao et al. [13] proposed a faster mean shift algorithm to solve the bottleneck of cell instance segmentation and tracking based on cosine embedding. This algorithm provides a plug-and-play model suitable for any clustering reasoning based on pixel embedding. At present, the digital construction of the smart grid provides massive data, while the development of deep learning provides an effective means for data value extraction. This also makes insulator-defect detection based on neural networks widely used.

The current research is mainly divided into two categories: the first is a two-stage object detection model represented by Region-based Convolutional Neural Networks (R-CNNs) [14], Fast Region-based Convolutional Neural Networks (Fast R-CNNs) [15], and Faster Region-based Convolutional Neural Networks (Faster R-CNNs) [16]. Lu et al. [17] proposed a Faster R-CNN based on an improved anchor box selection method to detect insulators. This method achieves good accuracy and takes less time to obtain the final result. However, it only detects three images per second, which is far from real-time detection. Liao et al. [18] proposed a Faster R-CNN algorithm combined with the deep residual network Resnet101 to detect insulator defects, which significantly improved the detection accuracy of insulator defects compared with traditional detection algorithms, but the algorithm has a large amount of calculation and cannot meet the real-time requirements of insulator-defect detection. The other category is the one-stage target detection model represented by You Only Look Once (YOLO) [19] and Single Shoot MultiBox Detector [20] for direct position regression. Feng et al. [21] proposed an automatic insulator detection method based on the YOLOv5 target detection model. Compared with four different versions of YOLOv5, the YOLOv5x model based on K-means clustering can effectively identify and locate insulator defects on transmission lines, but the highest accuracy rate is only 86.8%. Liu et al. [22] proposed the MTI-YOLO network, which uses a multi-scale feature detection head, a multi-scale feature fusion structure, and a spatial pyramid pooling model in the network, and which improves the accuracy of the model, but only detects normal insulators. Differing from the above two research methods, to realize end-to-end training and reasoning, Wu et al. [23] proposed an insulator-defect detection method based on Centernet, which simplified the backbone network and used an attention mechanism to suppress useless information and improve the accuracy of network detection. However, the detection speed is not high, and when two different kinds of objects share the same center point, CenterNet can only detect one of them.

Compared with the above algorithm, the YOLOv7 model proposed by Wang et al. [24] in 2022 has faster speed and higher accuracy on the COCO dataset. YOLOv7 includes several trainable bags of freebies so that the real-time detector can greatly improve detection accuracy without increasing the reasoning cost. It also studies how module re-parameterization can effectively replace the original module, and how the dynamic label allocation strategy can handle the allocation of different output layers. The speed and accuracy exceed all known target detectors in the range of 5 FPS to 160 FPS. It can also support both mobile GPUs and GPU devices from the edge to the cloud. In the future, the model can be deployed for practical engineering applications and meet the real-time requirements of insulator-defect detection. However, there is little research on the application of the YOLOv7 model to insulator-defect detection at present. At the same time, there is room for improvement in the accuracy of this model when detecting insulator defects. The accuracy of detection is also vulnerable to the impact of the complex background of transmission lines and small defect targets.

Aiming at the above problems, this paper proposes an improved insulator-defect detection method based on the YOLOv7 algorithm. Firstly, to improve the accuracy and efficiency of detection, this paper uses the anchor box size obtained from the K-means++ clustering insulator dataset to replace the default anchor box size of YOLOv7. Secondly, the coverage of overhead transmission lines is vast and the background of the collected image data is relatively complex. The deep-learning object-detection algorithm must be able to eliminate the interference of the complex background. In view of the complex background of image data, the CoordAtt attention mechanism and HorBlock module are integrated into the original backbone network to enhance the network’s ability to extract image features and increase the network’s detection accuracy for small insulator defect targets. The SIoU regression loss function and focal loss classification function are introduced to improve the network convergence speed and detection efficiency and solve the dataset’s sample imbalance problem. Finally, SIoU-NMS is used to implement a new non-maximum suppression process to reduce the problem of false detection of insulators and insulator defects. Experimental results show that the improved network has a better detection effect on insulator defects in complex environments.

## 2. Materials and Methods

### 2.1. Dataset Preparation and Preprocessing

#### 2.1.1. Image Acquisition

**Figure 1:**
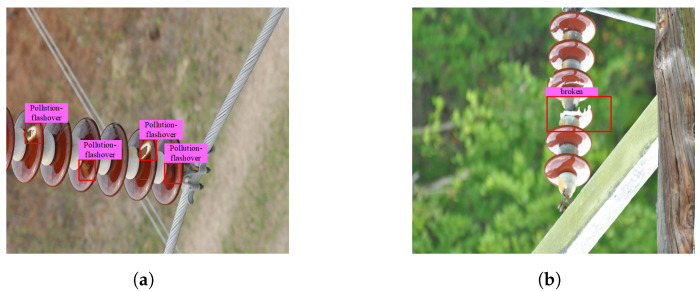
Two different types of defects: (**a**) pollution flashover; (**b**) damage.

There are few insulator-defect datasets published for overhead transmission lines, or even in the whole electric power field. In total, 1600 original images were captured from Baidu and Google or collated from public datasets [25]. The resolution width of the image pixels in this dataset is more than 2000–5000 and the height value is more than 2000–3000. Insulators and the insulator dataset are constructed according to the original pictures, which mainly include two defects: pollution flashover and damage, as shown in Figure 1.

#### 2.1.2. Image Preprocessing

**Figure 2:**
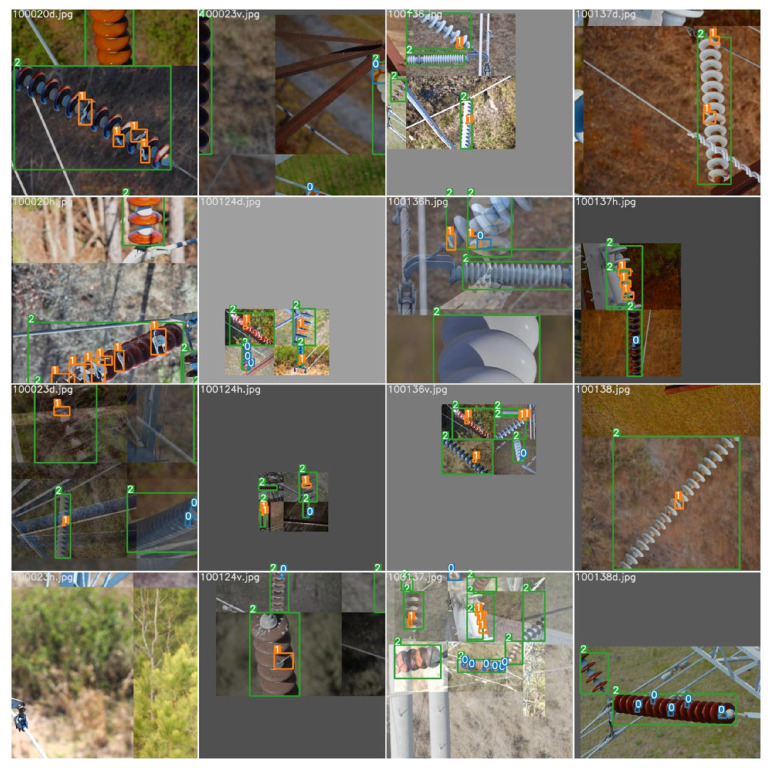
Renderings of data enhancements.

In a deep-learning model, numerous data samples are needed for model training to reduce the over-fitting problem. In the model-training stage, the more sufficient and comprehensive the data collected are, the more significant the model recognition effect. Therefore, the number of samples is expanded by data augmentation. The data augmentation strategy adopted in this paper includes morphological operations such as angle rotation, saturation adjustment, image up and down flipping, and translation. At the same time, the model uses the Mosaic data enhancement method at the input end. The four defect images are spliced by random scaling, random clipping, and random layout, which can improve the model classification performance. The mixup data enhancement method is also adopted and the two images are interpolated proportionally to mix the samples. Even color space conversion is carried out, and the pictures’ hue, saturation, and exposure are changed, in order to minimize the over-fitting of the network and improve the generalization ability of the training model. The input to the model after a series of operations is shown in Figure 2. 0 represents the defect of pollution flashover; 1 represents the defect of damage; 2 represents the insulator.

In addition to the above regularization techniques, this paper also adopts standard methods such as early stop, weight regularization, dropout, and batch normalization in the training phase to prevent overfitting of the model.

#### 2.1.3. Image Database and Label Database

**Figure 3:**
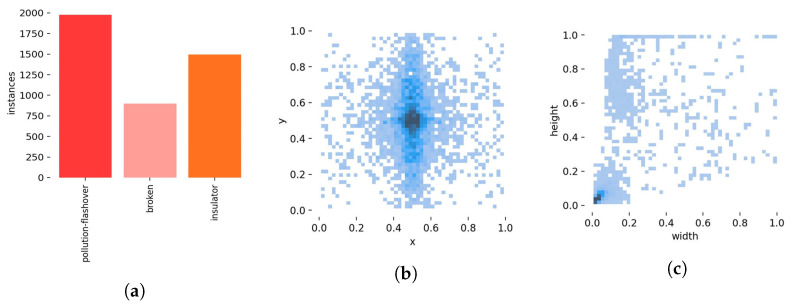
Labels and label distribution: (**a**) number of labels; (**b**) label location; (**c**) label size.

At the same time, the Labelme tool is used to label the ground-truth box of the image, and the label category is divided into pollution flashover, damage, and insulator. Finally, the labeled insulator dataset is divided into training, verification, and test sets. There are 1600 picture samples in this experiment and the dataset was divided at a ratio of 7:2:1. The number and distribution of tags in the dataset were counted; the results are shown in Figure 3.

In Figure 3a, the ordinate axis is the number of labels, and the abscissa axis is the name of labels. There are enough defect samples in the dataset for it to include most defect scenarios in insulators in daily life.

Figure 3b shows the distribution of tags. The abscissa x is the ratio of the abscissa of the label center to the image width, and the ordinate y is the ratio of the abscissa of the label center to the image height. As can be seen from the figure, the data is widely distributed and concentrated in the middle of the image. In Figure 3c, the abscissa width is the ratio of the label width to the image width, and the ordinate height is the ratio of the label height to the image height. The dataset contains data of various sizes, mainly small and medium target data, which is more suitable for the actual situation.

### 2.2. Proposed Methods

**Figure 4:**
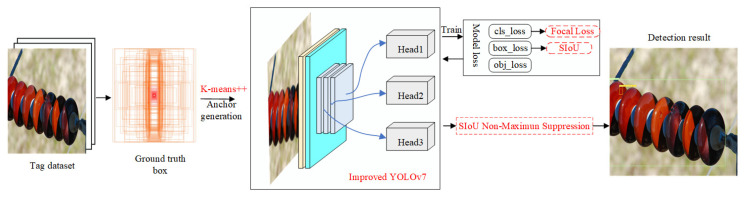
Detection framework of the proposed method.

The detection framework of the proposed method in this article is shown in Figure 4. Firstly, the insulator dataset is labeled with ground-truth boxes. Based on all ground-truth boxes in the dataset, anchor boxes with different sizes are generated by a clustering algorithm, which makes the initial anchor box size of the model match the target size of the insulator defects. Secondly, the network structure of YOLOv7 is changed. The CoordAtt attention mechanism and HorBlock module are added to the backbone network to focus on extracting helpful feature information in the image, while irrelevant features are weakened. Thirdly, the improved loss function is used to accelerate the convergence of the model. Finally, the SIoU NMS method is used to improve the non-maximum suppression process to reduce the multi-detection phenomenon of defect targets so that the model output results are more accurate.

### 2.3. Anchor-Box Optimization

YOLOv7 uses the K-means algorithm to cluster the anchor boxes obtained from the COCO dataset by default and uses the genetic algorithm to adjust the anchor boxes during the training process. However, the K-means clustering algorithm’s convergence depends heavily on the cluster center’s initialization [26]. Therefore, this paper uses the K-means++ algorithm [27] to alleviate this problem and improve the accuracy and efficiency of detection.

The traditional K-means algorithm selects multiple clustering centers at once, while the K-Means++ algorithm selects only one cluster center at a time. The improved method allows the random center point chosen to not just tend to the optimal local solution, but to come as close as possible to the optimal global solution.

Specific steps are as follows:

(1) Randomly select a sample target box from the dataset as the initial cluster center and calculate the minimum intersection ratio distance A(x) between the remaining sample boxes and the current cluster center.
  (1) $$\begin{array}{ccc} A(x) & = & 1-{\mathrm{IoU}}_{\left(x,c\right)} \end{array}$$
  where IoU represents the intersection parallel ratio between two rectangular boxes, *x* is the sub-target mark sample box, and *c* represents the center of the cluster;
(2) Calculate the probability O(x) that each insulator sample box is selected as the next cluster center and use the roulette method to select the next cluster center.
  (2) $$\begin{array}{ccc} O(x) & = & \frac{A{\left(x\right)}^{2}}{\sum_{x\in X}A{\left(x\right)}^{2}} \end{array}$$
  where *X* is the total sample of the target marker frame;
(3) Repeat Steps 1 and 2 until all clustering centers are selected.
(4) Calculate the distance from each sample in the dataset to the cluster centers, divide the sample into the class corresponding to the cluster center with the smallest distance, and recalculate the cluster center of each category $c_{i}$. Update the classification and cluster center repeatedly until the anchor box size remains unchanged.
  (3) $$\begin{array}{ccc} c_{i} & = & \frac{1}{\left|c_{i}\right|}\sum_{x\in c_{i}}x \end{array}$$
  where i=1,…,K, *K* is the number of anchor boxes with different sizes, and its value is determined by the number of anchor boxes of the detection model. Since the detection model in this paper contains three detection feature maps, and each feature map corresponds to three anchor boxes, k=9. The dimensions of the three feature maps and the corresponding optimized anchor boxes are shown in Table 1.

The small-sized anchor box corresponds to the final output of the 80 × 80 feature map, which is responsible for detecting small-sized objects. The medium-sized anchor box corresponds to a feature map with a size of 40 × 40, which is responsible for detecting medium-sized objects. The large-sized anchor box corresponds to a feature map with a size of 20 × 20, which is responsible for detecting large objects in the image.

### 2.4. Backbone Network

**Figure 5:**
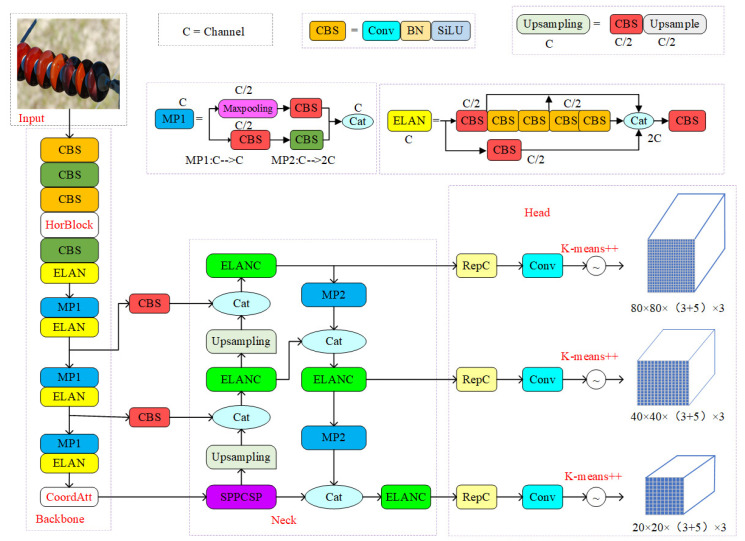
Structure of the improved YOLOv7 network.

The inspection model for insulator defects in this paper is shown in Figure 5. The whole network model structure is divided into four parts: input, backbone network, neck network, and head network.

First, the input side performs data enhancement operations such as Mosaic, random cropping, and scaling on the image to avoid overfitting. The backbone part comprises CBS, MP, and ELAN modules. The MP module consists of maxpooling and CBS modules. The CBS module consists of regular convolution, batch normalization, and activation functions. The difference between the CBS module and a traditional CNN network is that the Leaky ReLU function replaces the activation function with the SiLU function. The ELAN module can control the shortest and longest gradient paths, and deeper networks can learn and converge efficiently. The ELAN module is also composed of several CBS modules. The input feature map does not change the size of the feature map after passing through the ELAN module, but only changes the number of output channels at the end.

The improved network adds the HorBlock module between the third and fourth CBS modules in the backbone section. It adds the CoordAtt attention mechanism between the original backbone and neck sections. The improved network can make the model pay more attention to valuable content and locations in the input image samples. Feature information can be effectively extracted for small targets and targets with complex backgrounds to improve detection accuracy.

The neck consists of a path aggregation network PAN (path aggregation network) and a feature pyramid network FPN (feature pyramid network). After the 32-fold downsampling feature map output by the backbone passes through the SPPCSP module, the number of channels changes from 1024 to 512. Then, the feature map performs feature fusion according to the top-down strategy and the bottom-up method. The structure of PA-FPN efficiently fuses feature maps at different levels. Compared with YOLOv5, YOLOv7 replaces the CSP module with the ELANC module, and the downsampling becomes the MP2 layer. After the PAFPN network, the network’s output is three layers of feature maps of different sizes. Finally, the network outputs the prediction results through the RepC and Conv modules in the head part.

#### 2.4.1. HorBlock Module

**Figure 6:**
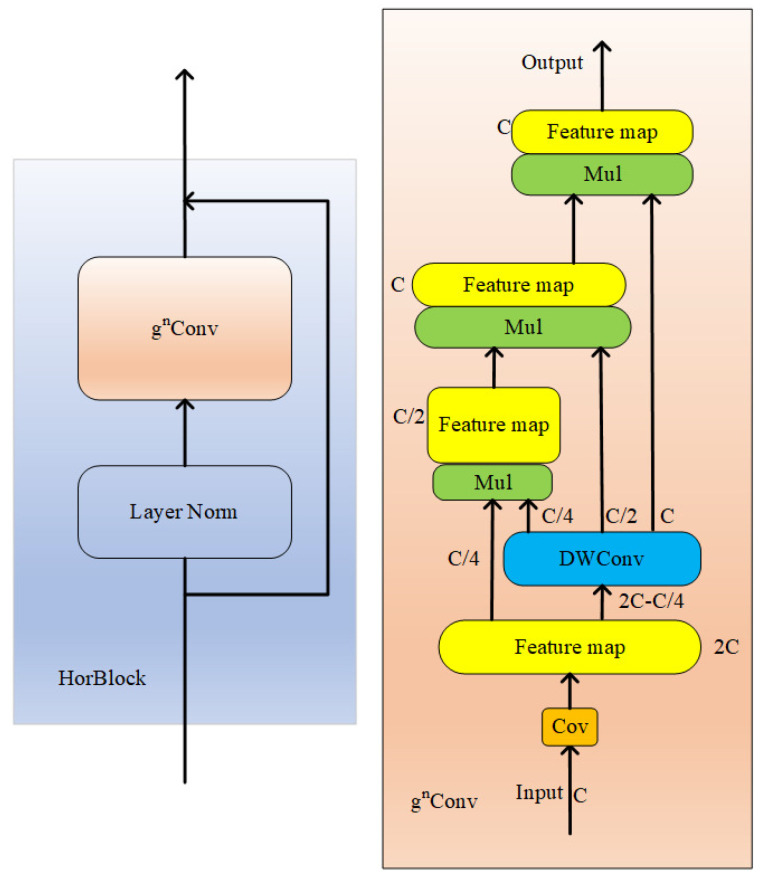
The schematic diagram of HorBlock.

After the network passes through the third CBS module of the backbone, the size of the feature map is further reduced by half and the feature information is greatly reduced. To retain the nonlinear ability, establish long-range attention, reduce the phenomenon of gradient dispersion, and improve the detection of insulator defects. HorBlock blocks consisting of gnConv [28] recursively gated convolution and layer norm normalization are added between the third and fourth CBS modules. The schematic diagram of the HorBlock block structure is shown in Figure 6.

The gnConv module is built using standard convolution, linear mapping, and element multiplication, but with input adaptive spatial blending capabilities similar to self-attention. Layer norm calculates the mean and variance of all parameters in all channels and then normalizes them. The main structure of gated convolution is not very different from a standard CNN, but the gating mechanism is introduced in the convolutional layer. First, the number of feature channels is adjusted by two convolutional layers. Next, the output feature map with separable convolution is divided into multiple parts, and each part is multiplied element by element to obtain the output feature map. Recursion here is the constant multiplication of elements, through which higher-order information can be saved.

#### 2.4.2. CoordAtt Module

Due to the complex and changeable environment in which the insulators are located, to improve the characteristic expression ability of the model on insulators and defective parts, an attention mechanism module is added to the end of the YOLOv7 backbone network.

Attention mechanisms can generally be divided into channel attention mechanisms, spatial attention mechanisms, and a combination of the two attention mechanisms. Traditional attention mechanism modules such as Squeeze-and-Excitation attention (SE) [29] and Convolutional Block Attention Module (CBAM) [30] have achieved good results in modeling channel-to-channel relationships, but are prone to spatial position information. Although the other attention modules without this problem have good effects, the number of parameters is too large and not suitable for application deployment.

The Coordinate Attention mechanism [31] captures not only cross-channel information but also directional perception and position perception, which helps the model more accurately locate and identify targets of interest. Second, the CoordAtt module is flexible and can be added in multiple places on existing models.

**Figure 7:**
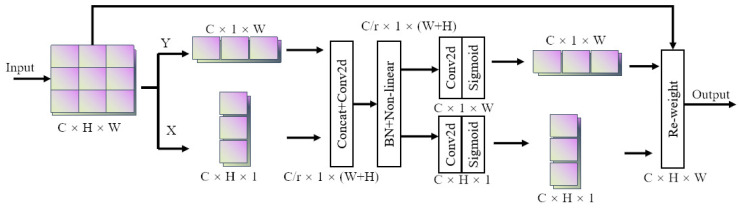
The schematic diagram of CoordAtt.

The CoordAtt attention mechanism framework diagram is shown in Figure 7.

The input is pooled horizontally and vertically to preserve the long-distance dependencies of both directions. The information in both directions is then stitched. Next, the feature map is split and convoluted to focus on both horizontal and vertical directions. The two-part feature map output by the module can be pinpointed to the row and column of the target object that we are interested in.

### 2.5. Loss Function

The loss function of the YOLOv7 model consists of three parts: localization loss ($L_{box}$), confidence loss ($L_{obj}$), and classification loss ($L_{cls}$). The total loss is the weighted sum of the three losses. Among them, the confidence loss and classification loss functions use binary cross-entropy loss, and the localization loss uses the CIoU loss function.

(4) $$\begin{array}{ccc} LOSS & = & W_{1}\times L_{\mathrm{box}}+W_{2}\times L_{\mathrm{cls}}+W_{3}\times L_{obj} \end{array}$$

where $W_{1}$, $W_{2}$, and $W_{3}$ are the weight values of the three loss functions, respectively. The loss functions optimized in this paper include regression and classified loss functions, which replace the traditional CIoU loss function with the SIoU regression loss function and use the focal loss classification loss function instead of the standard cross-entropy loss function.

#### 2.5.1. SIoU Loss

Traditional regression losses such as GIoU [32], DIoU [33], and CIoU [34] only consider the distance, overlapping areas, and aspect ratio of the prediction box and the ground-truth box, and do not take into account the angle between the ground-truth box and the prediction box, resulting in slower convergence. However, Gevorgyan [35] proposed the SIoU loss function. The SIoU regression loss function redefines the penalty metric by considering the vector angle between the desired regressions. This consideration can greatly speed up the training convergence process so that the prediction box first moves to the nearest axis (x-axis or y-axis). Then, the prediction box conducts regression along that axis.

The SIoU regression loss function consists of four parts: angle cost, distance cost, shape cost, and IoU cost. SIoU is defined as follows:

(5) $$\begin{array}{ccc} L_{box} & = & 1-\mathrm{IoU}+\frac{\Delta +\Omega }{2} \end{array}$$

(6) $$\begin{array}{ccc} x & = & \frac{\max\left(b_{cy}^{gt},b_{cy}\right)-\min\left(b_{cy}^{gt},b_{cy}\right)}{\sqrt{{\left(b_{cx}^{gt}-b_{cx}\right)}^{2}+{\left(b_{cy}^{gt}-b_{cy}\right)}^{2}}} \end{array}$$

(7) $$\begin{array}{ccc} \wedge & = & 1-2*\sin^{2}\left(\arcsin\left(x\right)-\frac{\pi }{4}\right) \end{array}$$

(8) $$\begin{array}{ccc} \Delta & = & 2-e^{\left(\wedge -2\right)\times {\left(\frac{C_{h}}{C_{h1}}\right)}^{2}}-e^{\left(\wedge -2\right)\times {\left(\frac{C_{w}}{C_{w1}}\right)}^{2}} \end{array}$$

(9) $$\begin{array}{c} \Omega ={\left(1-e^{-\frac{\left|w-w^{gt}\right|}{\max\left(w,w^{gt}\right)}}\right)}^{\theta }+{\left(1-e^{-\frac{\left|h-h^{gt}\right|}{\max\left(h,h^{gt}\right)}}\right)}^{\theta } \end{array}$$

(10) $$\begin{array}{ccc} IoU & = & \frac{A⋂B}{A\cup B} \end{array}$$

where IoU, Δ, and Ω are the intersection-over-union loss, distance loss, and shape loss, respectively. At the same time, the calculation of the distance loss takes into account the loss of the angles of the two boxes.

**Figure 8:**
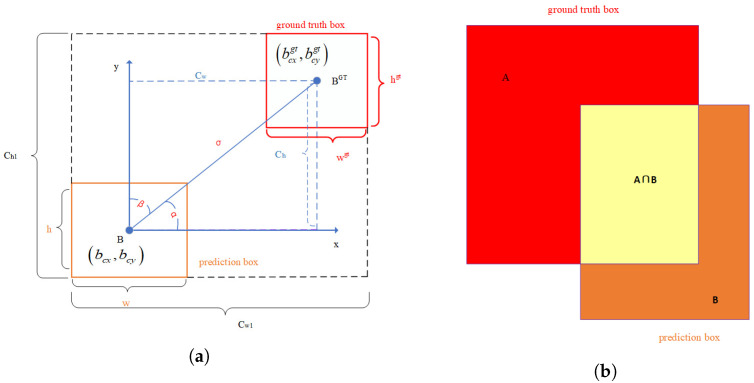
Schematic diagram of the calculation: (**a**) schematic diagram of the calculation of the angle cost; (**b**) schematic diagram of IOU calculation.

As shown in Figure 8a, $C_{w}$ and $C_{h}$ are the width and height of the rectangle constructed diagonally by connecting the center points of the two boxes, $C_{w1}$ and $C_{h1}$ are the width and height of the minimum bounding rectangle of the two boxes, $w^{gt}$ and $h^{gt}$ are the width and height of the ground truth box, and *w* and *h* are the width and height of the predicted box. α is the included angle between the line connecting the center point of the two boxes and the x-axis, and β is the included angle between the diagonal of the two boxes’ center points and the y-axis. θ is an adjustable variable, which indicates how much weight the network gives to the shape loss. The schematic diagram of IoU calculation is shown in Figure 8b, which calculates the ratio of the intersection and union of the ground-truth box and the predicted box.

The angle loss added to SIoU is mainly calculated for the distance loss between two boxes. Generally, in the early stage of model training, the predicted box and the ground-truth box do not intersect. Adding the loss of angle can speed up the calculation of the distance between the two boxes and make the distance between the two boxes converge quickly.

When the angle of α is greater than 45 degrees, the degree of β is used in the formula to replace the angle of α. At this time, the angle is considered from the x-axis to the y-axis. The network model will first try to make the center point of the predicted box parallel to the center point of the ground-truth box, and then let the predicted box continue to approach the ground-truth box along the relevant axis.

The traditional CIoU loss function converges with the overall shape of two boxes, while the SIoU regression loss function converges on two edges to achieve the effect of global shape convergence.

#### 2.5.2. Focal Loss

**Figure 9:**
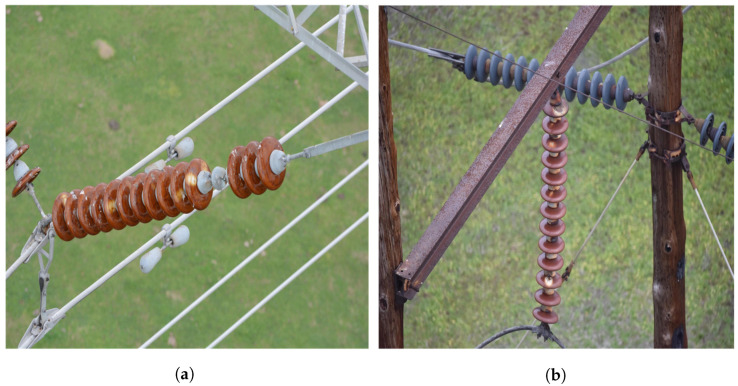
Samples with uneven complexity: (**a**) easily divided samples; (**b**) hard-to-classify samples.

The insulators and insulator defects in a picture sample are the foreground, while the other parts are called the background. The insulator image has an imbalance in foreground and background complexity, as shown in Figure 9. The number of insulators in Figure 9a is small and the background is simple. However, the number of insulators in Figure 9b is large and the background is complex. At the same time, there is an imbalance in the number of positive and negative samples in each category. The original network directly uses cross-entropy as the loss function to evaluate the model. However, as one-stage target detectors, the YOLO series has the problems of sample complexity and the unbalanced number of positive and negative samples [36], which will affect the gradient update direction of the network and lead to lower accuracy of final detection compared to a two-stage detector.

Given the above problems, the cross-entropy function is improved. The focal loss function can solve the imbalance between positive and negative samples in target detection. It is possible to add weight to the loss corresponding to the sample according to the difficulty of sample discrimination, that is, add less weight to the easily distinguishable sample and add greater importance to the complex distinguishable sample.

The formula for the classification loss function is as follows:

(11) $$\begin{array}{ccc} L_{\mathrm{cls}} & = & -\zeta_{t}{\left(1-p_{t}\right)}^{\delta }\log\left(p_{t}\right) \end{array}$$

(12) $$\begin{array}{c} p_{t}=\left\{\begin{array}{cc} p & \;\mathrm{if}\;y=1\\ 1-p & \;\mathrm{otherwise}\; \end{array}\right. \end{array}$$

(13) $$\begin{array}{c} \begin{array}{c} \zeta_{t}=\left\{\begin{array}{cc} \zeta & \;\mathrm{if}\;y=1\\ 1-\zeta & \;\mathrm{otherwise}\; \end{array}\right. \end{array} \end{array}$$

where $L_{cls}$ is the classification loss value and *p* is the probability that the sample predicted by the model belongs to the foreground. To solve the imbalance of sample categories, the weight parameter ζ is introduced. The adjustment factor δ is added to the cross-entropy loss function.

### 2.6. Non-Maximum Suppression

The traditional NMS [37] method has some defects in calculating IOU. If traditional NMS is used to screen the predicted box of two close objects, there will be missed detection.

The improvement of this paper is to introduce target scale and distance into the consideration of IOU, and use SIoU to calculate the IOU values of the candidate box with the highest confidence and all other boxes to determine which box to delete. It can solve the problem that of the insulator shield being too close to the insulator.

The improved non-maximum suppression algorithm of SIoU-NMS is used to filter the preliminary prediction box of the image output to be recognized, and the final prediction box is obtained by the following steps:

(1) Set the confidence threshold and SIoU threshold;
(2) Calculate the confidence level of all preliminary prediction boxes output by the network model, put the preliminary prediction boxes whose confidence level is higher than the confidence threshold in the candidate list, and sort the preliminary prediction boxes in descending order of confidence from high to low in the candidate list;
(3) Take the initial prediction box with the highest confidence from the candidate list, save it to the output list, and delete the initial prediction box from the candidate list;
(4) Calculate the cross-merger loss of the initial prediction box with the highest confidence obtained in the previous step and all the other preliminary prediction boxes in the candidate list, and delete the initial prediction box whose cross-merger loss is higher than the set SIoU threshold from the candidate list;
(5) Repeat Steps 3 and 4 until the candidate list is empty;
(6) Use the preliminary prediction box in the output list as the final prediction box.

## 3. Results and Discussion

### 3.1. Experimental Environment

To verify the effectiveness of our model, we conducted neural network training and testing with the following computer-configuration parameters and super-parameter settings (shown in Table 2).

### 3.2. Visual Analysis of Model

**Figure 10:**
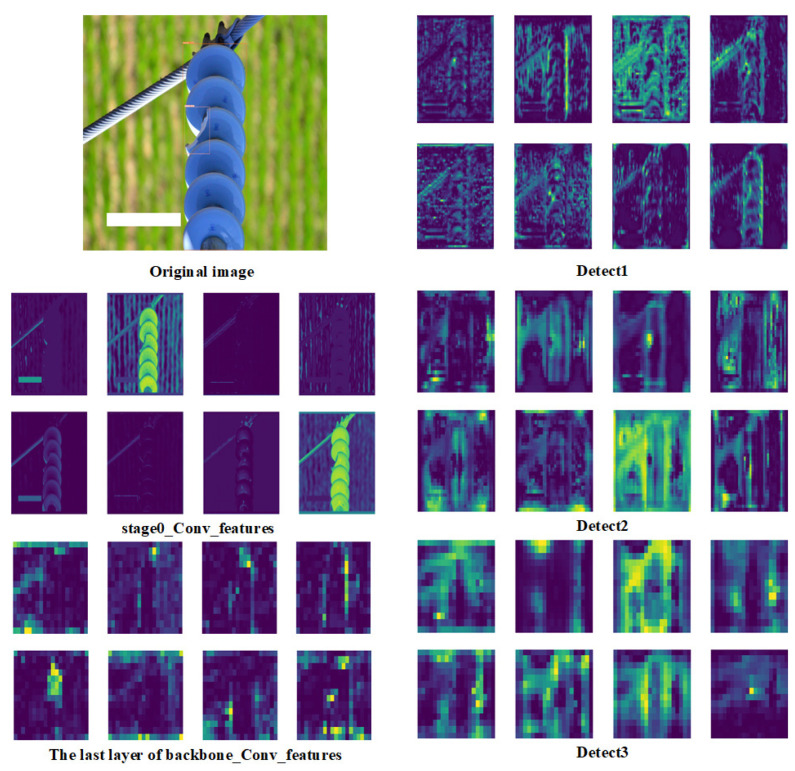
Visualization of the feature map.

After training the model, we visualized the feature map of the trained model [38]. The information that the network model is interested in can be seen from the visualized feature map. In addition, we further examined whether the model contributed to the attention mechanism. Figure 10 shows the feature diagram of the first convolution module visualized by the model, the backbone module, and the output of the three detectors.

From the visual feature map after the first convolution layer, we can see that the features extracted from the model have particular emphasis; some focus on edge features and some on overall features. Of course, this is only the feature map after the first convolution layer. Compared with the deeper features, the shallow features are mostly complete, while the deeper network features will be fewer. From the feature map output after the backbone, we can see that adding an attention mechanism will play a good role in strengthening the feature map and suppressing some unnecessary features. The feature maps corresponding to the last three layers are used to detect large, medium, and small targets, which significantly improves the multi-scale detection capability of the model.

**Figure 11:**
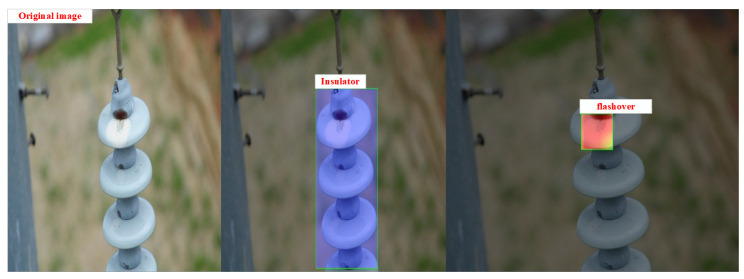
Grad-CAM visualizations for the original categories.

After the target is detected, the model also needs to carry out classification tasks. Figure 11 shows the class activation map of the model, which can further visualize which pixels of the image the neural network pays attention to when predicting a certain category. It can be seen that the improved algorithm can better extract the target feature information from the insulator image.

### 3.3. Evaluation Metrics

To comprehensively and objectively evaluate the performance of our model in this paper, we used a confusion matrix (Shown in Table 3) for comprehensive evaluation.

TP represents correct detection—the prediction of the model is positive and the actual value is also positive. FN represents detection error—the prediction of the model is negative and the actual value is positive. FP represents detection error—the prediction of the model is positive but the actual value is negative. TN represents correct detection—the model prediction is negative and the actual value is also negative.

The expressions of precision and recall are as follows:

(14) $$\begin{array}{c} \;\mathrm{Precision}\;=\frac{\mathrm{TP}}{\mathrm{TP}+\mathrm{FP}} \end{array}$$

(15) $$\begin{array}{c} \;\mathrm{Recall}\;=\frac{\mathrm{TP}}{\mathrm{TP}+\mathrm{FN}} \end{array}$$

Mean average precision (mAP), which depends on precision and recall, is used to measure the accuracy of the model.

### 3.4. Experimental Results and Analysis

To verify the effectiveness of the improved method proposed in this paper, we performed comparative experiments on the setting of super-parameters of the anchor box, the selection of the loss function, and the selection of attention mechanisms. The baseline of each experiment is the YOLOv7 model. Because this paper’s primary goal is to improve detection accuracy, our experiment here mainly uses recall, precision, and mAP to evaluate the effect.

First, we determined the nine parameter values of the anchor box. For the generation of the anchor box, we used the K-means algorithm to cluster the anchor box of the COCO dataset and K-means++ clustering to compare the parameters. It can be seen from the results (shown in Table 4) that all the evaluation indexes have increased significantly when using the nine anchor frame parameters obtained after K-means++ clustering, which verifies the idea of selecting the initial center of the anchor box with appropriate methods.

Then, we made a comparison of different loss functions. YOLOv7 uses CIoU as its loss function. We compared the performances of three methods: GIoU, CIoU, and SIoU (ours); the results are shown in Table 5.

As seen from Table 5, using SIoU can improve precision and recall by about 1.4% and 1.1%, compared with other methods. These experimental results show that the SIoU loss function can obtain good performance in insulator-defect detection.

In order to add appropriate attention mechanisms to the network, this paper added SE, CBAM, and CoordAtt attention mechanisms to the backbone layer and feature fusion layer of the model for training and comparison. The results are shown in Table 6.

The effect on model detection caused by different attention mechanisms added to the backbone is different. Not every kind of attention will improve the model’s performance after joining the network. Although SE attention ignores location information, it also considers channel attention. From the results, the performance effect of the model is reduced after SE attention is added to the backbone. CBAM tries to use location information by reducing the channel dimensions of the input tensor and then calculating spatial attention by convolution. However, convolution can only capture local relationships; it cannot model long-term dependencies crucial to visual tasks. After CBAM attention is added to the backbone, the performance effect of the model is improved, but it is not apparent. The CoordAtt attention mechanism embeds location information into channel attention, capturing remote dependencies in one spatial direction and keeping accurate location information in another. According to the results, the mAP increased by 1% after adding CoordAtt to the backbone. This proves that using the CoordAtt attention mechanism can make the network model notice the target in a broader range and improve the detection ability of the network.

Ablation experiments were carried out to verify the positive impact on the network of the improved strategy proposed in this paper. The improved strategy proposed in this paper was trained on the insulator dataset. The experimental ablation results are shown in Table 7 below. “✓” indicates that the corresponding improvement method is used, and “✕” indicates that the improvement method is not used.

The first row in the table is the detection result of the original YOLOv7 network. It can be seen from the above table that when the HorBlock module and CoordAtt module are added to the original network, mAP@0.5 and accuracy are increased by 1.3% and 1.1%, respectively, compared with the original YOLOv7 without any improvement strategy. When YOLOv7 uses the K-means++ algorithm to cluster anchor boxes generated by insulator datasets, the positioning accuracy is improved to 3.6% higher than the original YOLOv7 algorithm. In addition, because the K-means++ algorithm does not increase the number of layers and parameters of the network, it will not reduce the model’s speed. Moreover, K-means++ does not have the problem of significantly affecting the initial value selection, which can improve the speed of model border regression. Using the improved strategy of SIoU+NMS, precision increased by 2.4% and mAP@0.5 increased by 1%.

Combining the above three groups of improved methods, it can be found that the combined improved algorithm has the best effect; the detection accuracy can reach 94.9% and mAP@0.5 can reach 93.8%, which can meet the requirements for detection accuracy on insulator images.

Table 8 shows the detection of this model in each category. The insulator has high precision and low leakage rates. However, because complex environments and tiny targets characterize the defects of pollution flashover and damaged insulators, the precision and recall rates are not as good as those of insulators.

Precision and recall cannot be used as the only indicators to measure the model’s performance, as they may lead us to misunderstand the model’s performance. Therefore, we further use a PR curve to measure the model’s performance. The PR curve comprehensively considers the precision and recall rate of each category detected by the model.

**Figure 12:**
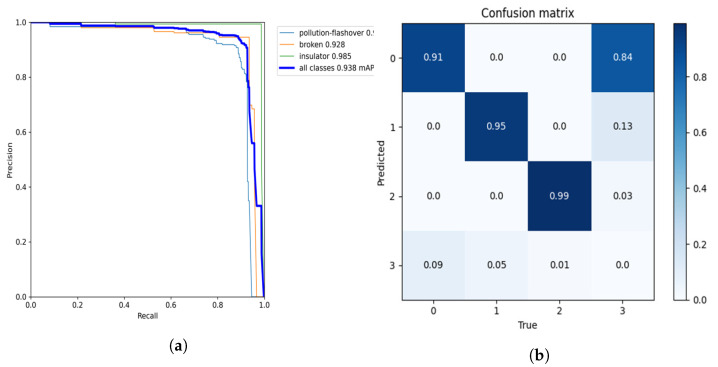
Operation result curves of this model: (**a**) precision–recall curve; (**b**) confusion matrix.

The PR curve of this model is shown in Figure 12a. The horizontal axis of the PR curve is recall and the vertical axis is precision. You can intuitively see the change rate of precision as recall increases. If the curve in the figure is close to the upper right corner, it means that with the increase in recall, the fall in precision is not obvious, and the overall performance of the model is better. Figure 12b is a confusion matrix diagram; 0 represents the defect of pollution flashover; 1 represents the defect of damage; 2 represents the insulator; 3 represents the background. The row direction in the figure represents the real label and the column direction represents the predicted category. From the values in each line, the correct detection rates of damage and pollution flashover are 95% and 91%. The confusion matrix is a summary of the prediction results of classification problems. It can be seen that the classification of insulator defects is accurate.

The ablation experiments can only prove that the improved strategy in this paper is effective compared with the original algorithm, but whether it can reach an advanced level needs to be proved. Therefore, under the same experimental conditions, a series of comparative experiments were carried out on insulator datasets between the improved method and the current mainstream target detection method.

**Figure 13:**
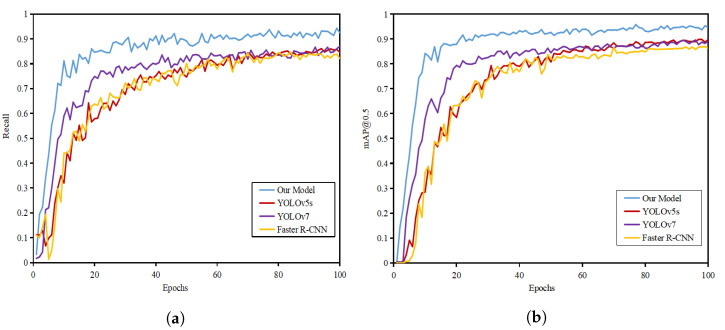
Comparison of training results of different models: (**a**) recall curve; (**b**) mAP@0.5 curve.

The comparison of the training results of different models is shown in Figure 13. From the figure, it can be seen that the mAP@0.5 and recall of the improved algorithm in this paper are significantly higher than those of the other three models.

**Figure 14:**
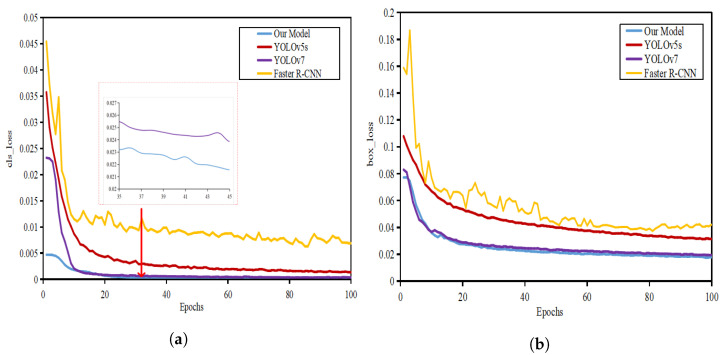
Comparison of training loss curves of different models: (**a**) cls-loss curve; (**b**) box-loss curve.

Figure 14 shows the comparison of training loss curves of different models. After 20 iterations, the loss curves of different models are stable and the training results can converge. It can be seen that YOLOv5s is far less effective than YOLOv7 in terms of regression loss and classification loss. Since the network structure is added to the improved model in this paper, the convergence speed of the improved model in the figure is slightly lower than that of YOLOv7. However, in about 20 iterations, the model in this paper shows a better decline rate and convergence ability than YOLOv7. It is proven that the loss function’s adjustment improves the network’s convergence ability.

**Figure 15:**
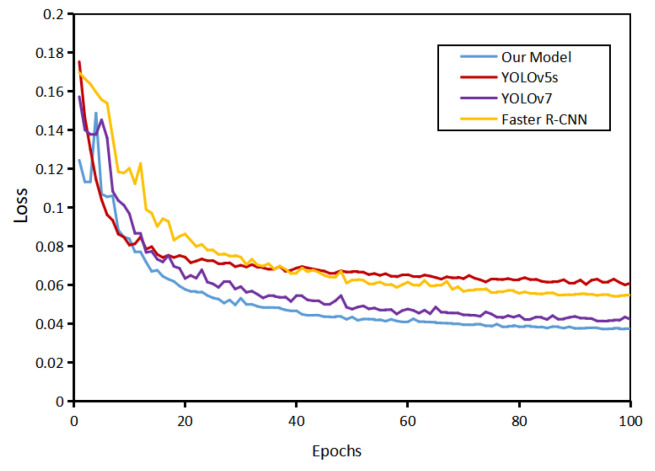
Comparison of model loss curves on the testing data.

Compare the loss function curves during model training; Figure 15 shows the loss on the test set. The loss is the sum of model regression, confidence, and classification loss. The figure shows that the effect of our model is better than other models.

Finally, Table 9 lists the comparison results of evaluation indicators of different models. It can be seen from the table that the recall of this model was greater by 6.3% and mAP@0.5 by 3.7% compared with YOLOv7. Although the model’s speed has decreased, the speed is still 95 FPS, much faster than the speed of the two-phase model.

Based on the comparison and analysis of the above series of experiments, it can be concluded that the improved YOLOv7 algorithm proposed in this paper has obvious advantages in detection accuracy. Although the speed is decreased to a certain extent, it can still meet the real-time requirements of insulator-defect detection in practical engineering.

**Figure 16:**
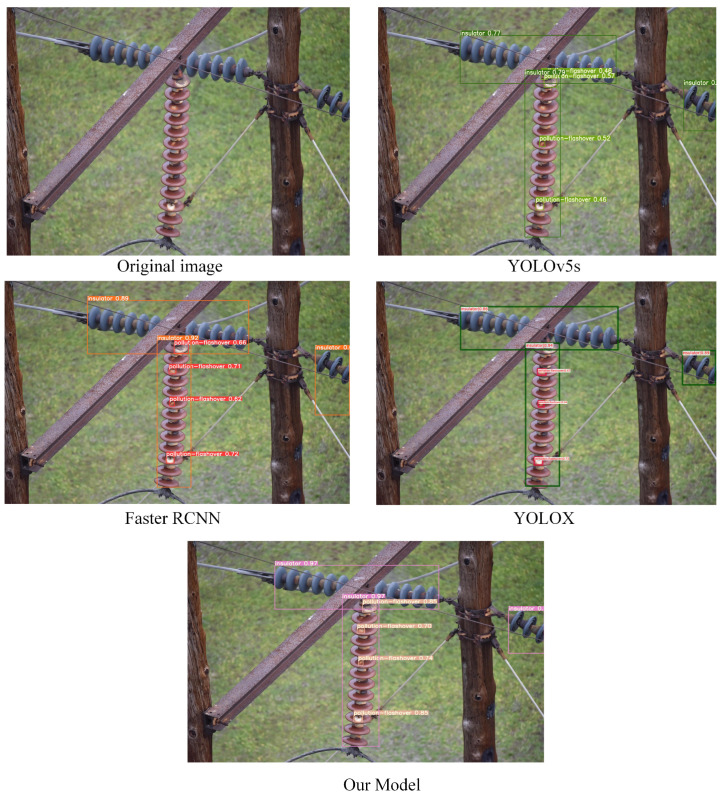
Detection results after the target are obscured.

**Figure 17:**
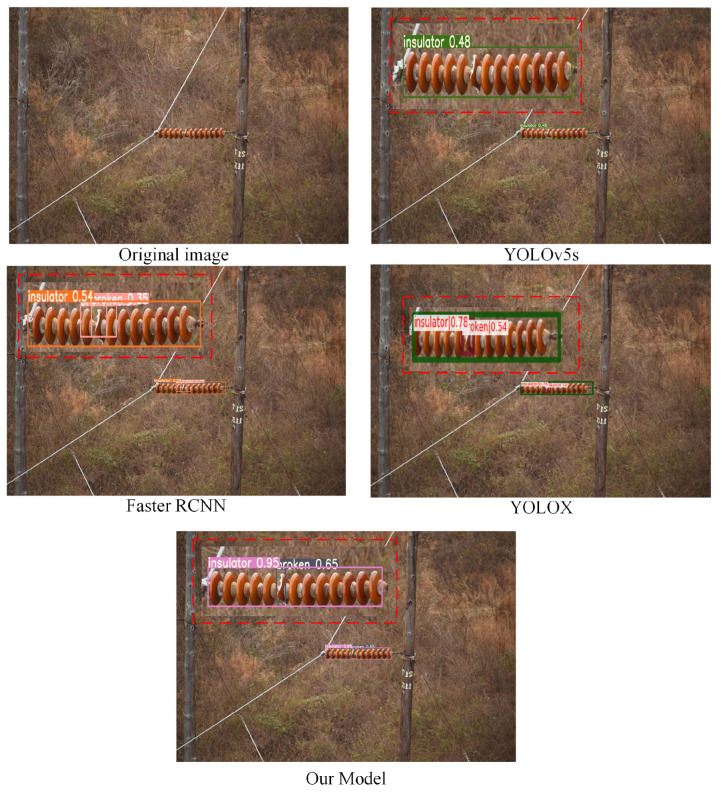
Small-object detection experimental results.

**Figure 18:**
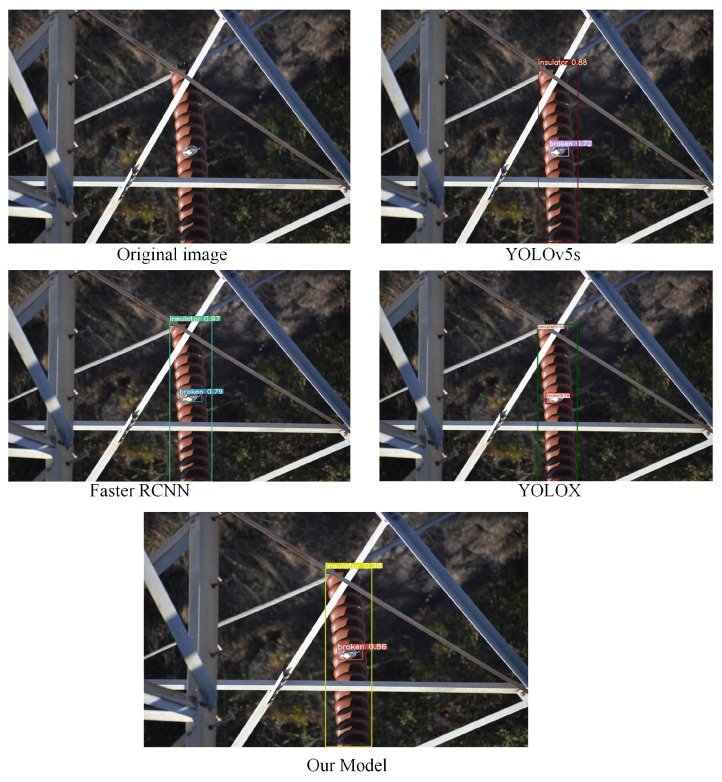
Detection results of images with obscured objects and complex backgrounds.

To better verify the generalization ability and robustness of the model in this paper, we specifically selected small targets and targets in complex environments in the test set for testing. In the contrast-detection experiment, to verify that the model in this paper is more suitable for insulator-defect detection, we also purposely add the comparison of detection results of the YOLOX model. The detection results are shown in Figure 16, Figure 17 and Figure 18. Through comparative analysis, the improved algorithm can better identify micro-defects of insulators and can accurately identify some insulator targets in complex environments.

In conclusion, the results of ablation and comparison tests show that the strategy in this paper has significantly improved the accuracy of insulator-defect detection.

Dian et al. [39] previously proposed a Faster R-Transformer algorithm for aerial insulator detection. The algorithm also combines self-attention mechanism, with an average accuracy of 97.31%. However, the FPS is only 12, which does not meet the actual application requirements of the project. The research results in this paper can improve the accuracy, while the FPS is still 95. Ding et al. [40] detected insulator defects by improving the classic YOLOv5 model. This model also improves the regression loss function, anchor frame generation method, and NMS method, which improves the model detection accuracy. However, the recall rate is only 90.4%, while the recall rate in this paper is 93.4%.

The improved strategy proposed in this paper provides more possibilities for small-target detection such as insulator defects. In the future, this model can also be applied to various small-target detection scenarios in agriculture and industry. Finally, the improvement of this paper is mainly in the backbone of the network. The detection head is also important for model fusion features. We plan to expand our research in the future to understand the network model more fully.

## 4. Conclusions

This paper proposes an improved YOLOv7 insulator-defect detection method, which can accurately identify insulators and their defects in transmission line images with complex backgrounds. The experimental analysis proves that the anchor box generated by clustering the target box of the insulator dataset with K-means++ can improve the detection accuracy and effect. Combining the CoordAtt attention mechanism and HorBlock module in the network can increase the expression ability of the feature graph, optimize the feature representation of the insulator-defect target, and improve the detection effect of the insulator-defect target. Finally, SIoU, the loss function, introduces the concept of the angle between the real box and the predicted box to help calculate the distance between the two boxes, accelerating the network’s convergence. By adding the above strategies, the improved model increases recall and mAP@0.5 by 2.5% and 2.7%, respectively, compared with the original network. In addition, the detection speed of the model is still 91 fps, which can meet the needs of real-time high accuracy. Compared with other models, this method has obvious advantages.

There are still some improvements to be made. At present, the insulator defects in the dataset are external contour damage, and there is no image dataset of internal crack defects of insulating materials. Suppose that an internal crack defect in the insulating material shows some characteristics on the outer surface of the insulator. We will further improve the dataset to detect insulator defects in that case.

## Figures and Tables

**Table 1:** Optimized anchor box parameters.

Feature Map Size	80 × 80	40 × 40	20 × 20
YOLOv7	(13, 12)	(135, 131)	(430, 112)
	(21, 21)	(65, 326)	(188, 324)
	(39, 31)	(101, 304)	(393, 266)

**Table 2:** Experimental environment configuration.

Parameter	Configuration
CPU	12th Gen Intel(R) Core i9-12900KF 3.2 GHz
GPU	NVIDIA GeForce RTX3080 Ti (12G)
Operating System	Windows10
CUDA	11.3
Python	3.7.13
Torch	1.10.0
Momentum	0.937
Weight decay	0.0005
Batch size	16
Learning rate	0.01
Image size	640 × 640
Epochs	100

**Table 3:** Confusion matrix.

	Prediction	Positive	Negative
Reference			
Positive		True positive (TP)	False negative (FN)
Negative		False positive (FP)	True negative (TN)

**Table 4:** Performances of different anchor boxes parameters.

Anchor Boxes Parameters	Precision	Recall	mAP@0.5
Initial anchor box	0.9	0.871	0.901
Clustered anchor box (ours)	0.911	0.884	0.914

**Table 5:** Performances of different loss function.

Loss Function	Precision	Recall	mAP@0.5
GIoU	0.895	0.867	0.9
CIoU	0.9	0.871	0.901
SIoU (ours)	0.914	0.882	0.905

**Table 6:** Effects of models with different attention mechanisms.

Strategy	mAP@0.5	FPS
Backbone	0.901	107
Backbone+SE	0.897	105
Backbone+CBAM	0.906	103
Backbone+CoordAtt (ours)	0.911	102

**Table 7:** Ablation experiment results.

Model	HorBlock+CoordAtt	K-Means++	SIoU+NMS	mAP@0.5/%	Precision/%	FPS (Frames per Second)
YOLOv7	✕	✕	✕	90.1	90	107
	✓	✕	✕	91.4	91.1	101
	✕	✓	✕	90.3	93.6	108
	✕	✕	✓	91.1	92.4	104
	✓	✓	✓	93.8	94.9	95

**Table 8:** Detection effect of the model on each category.

Class	Precision	Recall	mAP@0.5
Pollution flashover	0.92	0.87	0.9
Damage	0.94	0.93	0.93
Insulator	0.97	0.98	0.99

**Table 9:** Comparison of evaluation indicators of different models.

Method	F1 Score	Recall	mAP@0.5	FPS
Faster R-CNN	0.80	0.821	0.862	12
YOLOv5s	0.89	0.845	0.898	111
YOLOv7	0.88	0.871	0.901	107
Ours	0.94	0.934	0.938	95

## Data Availability

Dataset link: https://doi.org/10.6084/m9.figshare.21200986 (accessed on 24 September 2022).
